# Polydopamine-Modified Liposomes: Preparation and Recent Applications in the Biomedical Field

**DOI:** 10.1021/acsomega.4c02555

**Published:** 2024-05-28

**Authors:** Anna Maria Maurelli, Vincenzo De Leo, Lucia Catucci

**Affiliations:** † Department of Chemistry, University of Bari Aldo Moro, Via Orabona 4, 70126 Bari, Italy; ‡ CNR-IPCF S.S. Bari, c/o Department of Chemistry, University of Bari Aldo Moro, Via Orabona 4, 70126 Bari, Italy

## Abstract

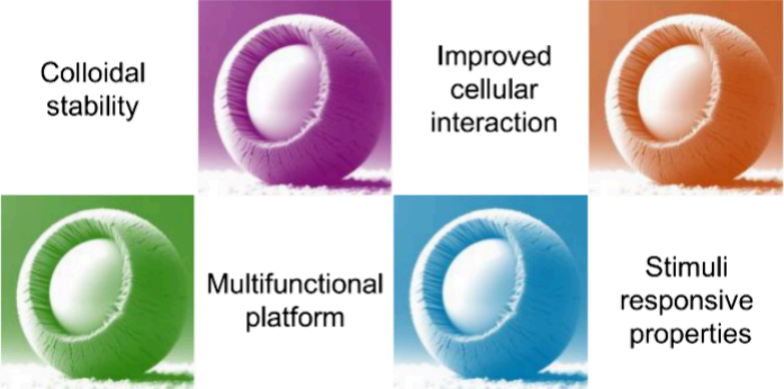

Polydopamine (PDA) is a bioinspired polymer that has unique and desirable properties for emerging applications in the biomedical field, such as extraordinary adhesiveness, extreme ease of functionalization, great biocompatibility, large drug loading capacity, good mucopenetrability, strong photothermal capacity, and pH-responsive behavior. Liposomes are consolidated and attractive biomimetic nanocarriers widely used in the field of drug delivery for their biocompatibility and biodegradability, as well as for their ability to encapsulate hydrophobic, hydrophilic, and amphiphilic compounds, even simultaneously. In addition, liposomes can be decorated with appropriate functionalities for targeted delivery purposes. Thus, combining the interesting properties of PDA with those of liposomes allows us to obtain multifunctional nanocarriers with enhanced stability, biocompatibility, and functionality. In this review, a focus on the most recent developments of liposomes modified with PDA, either in the form of polymer layers trapping multiple vesicles or in the form of PDA-coated nanovesicles, is proposed. These innovative PDA coatings extend the application range of liposomes into the field of biomedical applications, thereby allowing for easier functionalization with targeting ligands, which endows them with active release capabilities and photothermal activity and generally improves their interaction with biological fluids. Therefore, hybrid liposome/PDA systems are proposed for surface-mediated drug delivery and for the development of nanocarriers intended for systemic and oral drug delivery, as well as for multifunctional nanocarriers for cancer therapy. The main synthetic strategies for the preparation of PDA-modified liposomes are also illustrated. Finally, future prospects for PDA-coated liposomes are discussed, including the suggestion of potential new applications, deeper evaluation of side effects, and better personalization of medical treatments.

## 1 Introduction

In recent years, polydopamine (PDA), its related derivatives, and its (nano)composites have attracted widespread attention. Although the full understanding of the mechanism behind the formation of PDA from dopamine (DA) monomers remains an open challenge, numerous applications for the PDA have been proposed, and numerous materials have been enriched with surface coatings based on this polymer for the most disparate purposes. In fact, the phenolic and amino moieties of the PDA polymer synergistically contribute to adhesion on virtually any material surface, which dramatically changes its properties.

PDA has been used to coat colloidal particles designed for biomedical purposes, in particular, as systems for drug delivery applications. Among these, liposomes have been used in different arrangements to obtain surfaces or nanoparticles with high biocompatibility, controlled drug release properties, ease of targeting, photothermal capacity, etc.

Although the properties and applications of liposomes, including polymer-modified liposomes, have been extensively reported in the literature, this review focuses in particular on a specific and emerging area of research in which PDA, a bioinspired polymer rich in unique features, is used to enrich liposomes in order to extend their applications in new directions, as illustrated in Sections 3.1–3.7. Thus, in this review, the main properties of PDA in the biomedical field and those of liposomes will first be introduced. Subsequently, the ameliorative effects conferred by PDA coatings to liposomes and the emerging properties and application possibilities of these hybrid constructs in different arrangements will be discussed with reference to the most up-to-date literature. The preparation strategies of PDA-modified liposomes will also be briefly illustrated. Finally, some suggestions will be presented to overcome current limitations to the application of PDA-modified liposomes in clinical practice and explore new possibilities.

## 2 Polydopamine Overview

### 2.1 PDA Structure and Synthesis

**Figure 1:**
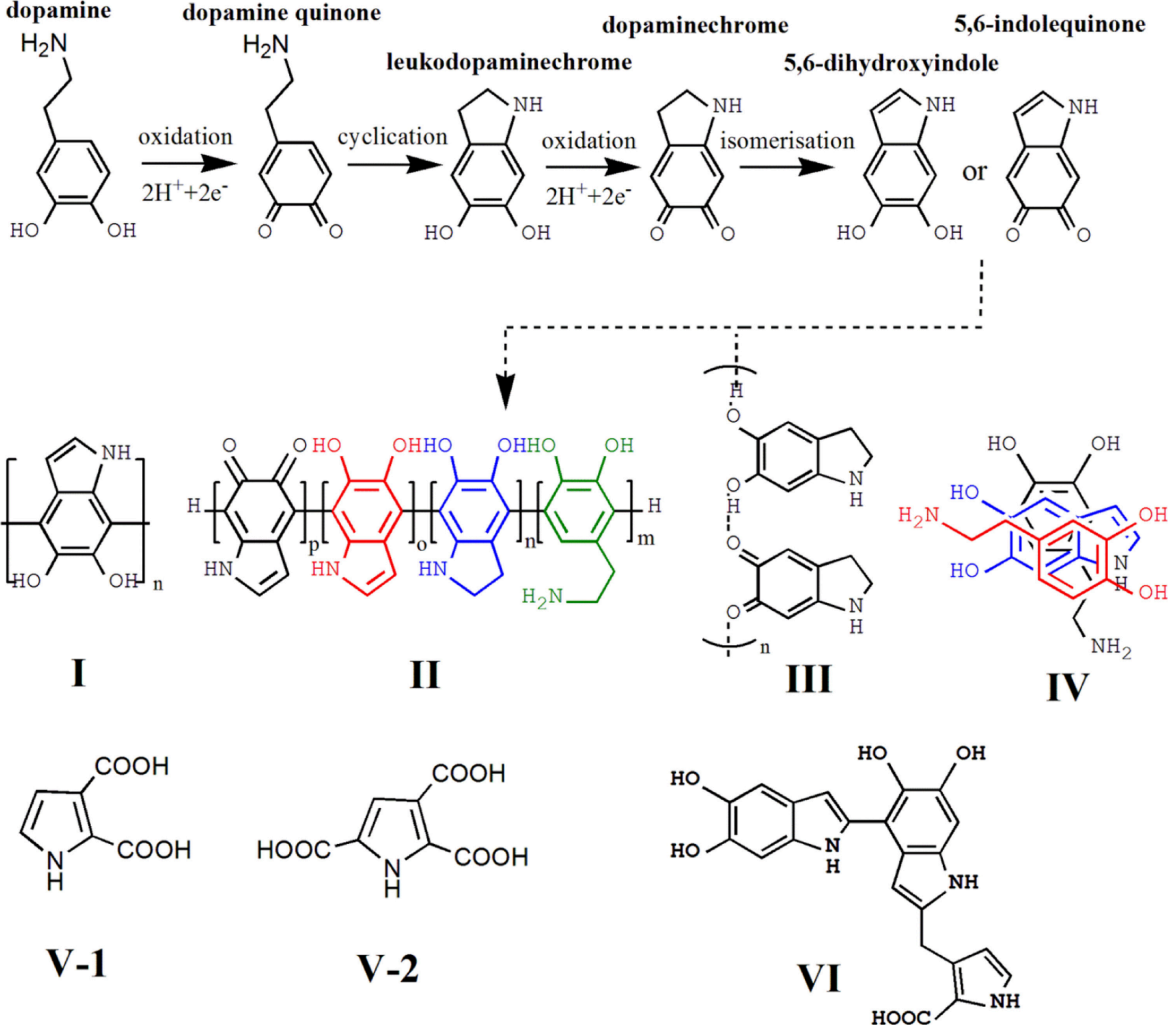
Illustration of the first steps of the mechanism of dopamine oxidation to form PDA and possible derived structures. Reproduced from ref (3) under Creative Commons license.

Generally, “polydopamine” is intended as the product of the self-oxidation and self-polymerization of DA, which can be conducted under a variety of experimental conditions.^1^ Indeed, the PDA can be obtained throughout the self-polymerization of the DA in water under oxygenated and slightly alkaline conditions or by electro-polymerization, UV irradiation, enzyme-assisted polymerization, etc.^2^ In addition, the polymerization reaction can be performed to obtain PDA-only structures or, more commonly, to realize composite structures in which PDA forms films or coatings on the most disparate materials. Although PDA has been the object of plenty of studies and applications, its effective polymeric structure has not yet been fully elucidated, also because this varies depending on the particular polymerization conditions. Generally speaking, it appears to be similar to l-DOPA, which is found in the byssus secreted by mussels, but it is strongly debated whether PDA is a covalent polymer or the result of a noncovalent assembly of low-molecular-weight DA derivatives. Also, establishing a unique mechanism of PDA formation would be impossible considering that it, as well as its structure, strongly depends on the specific experimental conditions. When PDA is exploited for biomedical applications, polymerization is mostly performed in solution under alkaline conditions. In this case, similarities have been observed between the pathways of PDA formation and melanin biosynthesis.^3^ Several authors agree that the first steps involve the oxidation of dopamine to dopamine quinone, its intramolecular cyclization to leucodopaminechrome, and further oxidation to dopaminechrome, which leads to the formation of 5,6-dihydroxyindole (DHI) or 5,6-indolequinone (IDQ)^3^ (Figure 1). Then, the DHI can encounter covalent or noncovalent pathways to generate the PDA, also in relation to whether or not polymerization takes place in the presence of a substrate.^4^ Thus, the structure of the PDA seems to be both the results of covalent and noncovalent interactions among oxidated derivates of dopamine, but some recent evidence supports a predominantly covalent polymeric nature.^1^

Regarding the morphology of PDA-based structures arising in a simple alkaline aqueous environment, it has been reported that the polymerization of DA in the absence of templating agents, organic solvents, and other reagents usually leads to the formation of large PDA particles or PDA films that adhere to the walls of reaction vessels. If templating agents [i.e., nanoparticles (NPs)] are introduced into the reaction environment, the polymerization takes place predominantly on their surface, thereby easily giving rise to nanostructured systems with uniform size distributions.^5^

### 2.2 PDA Properties

**Figure 2:**
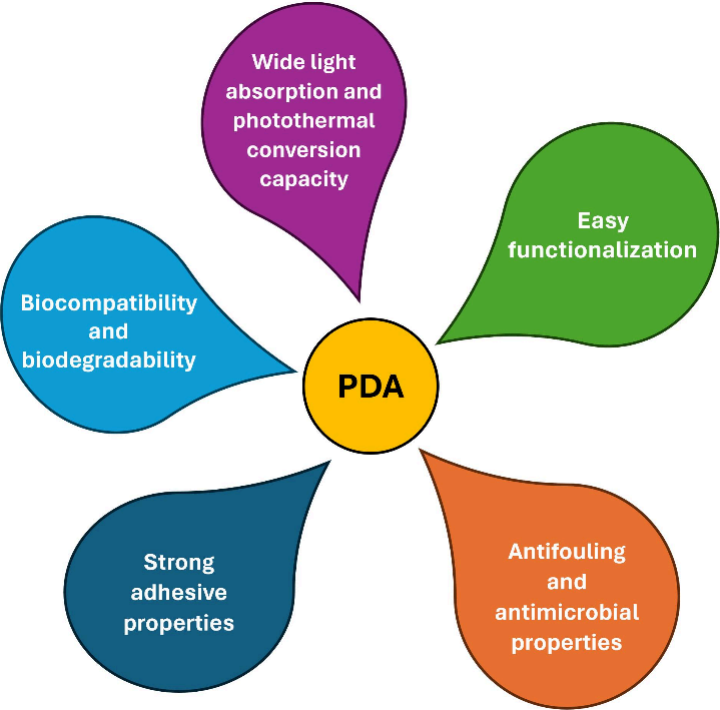
Main properties of the PDA.

From its discovery by Messersmith et al. in 2007,^6^ the use of the PDA has significantly spread in the literature. The easiness of obtaining coatings on different substrates, coupled with its fascinating properties, has prompted the use of PDA in the most disparate fields, such as biomedicine, biosensing and bioelectronics, environmental remediation,^2,5^ etc. The main properties of PDA are summarized in Figure 2. PDA is considered an extremely potent and universal adhesive material thanks to its capability to adhere to the surface of organic and inorganic substrates of different shapes and dimensions, even underwater. This represents a unique advantage as underwater adhesion is usually challenging because of the existence of a thin layer of hydration that hampers proper contact between a polymer and a substrate. The mechanism of adhesion of PDA is not fully understood, but it is generally recognized that the presence of catechol and amino groups contributes to the process. In addition, the presence of a large variety of functional groups, such as amino, catechol, and aromatic moieties, allows an easy further functionalization of PDA through covalent and noncovalent interactions.^6^ It can easily covalently react with thiol and amine groups through Michael addition and/or Schiff base reactions^7^ and weakly bind other structures through H-bonds, electrostatic and π–π stacking interactions.^1^ Moreover, PDA has a broad optical absorption and a high photothermal conversion efficiency attributable to the presence of conjugated systems and electron donor–acceptor pairs in the polymeric structure, possesses antifouling and antibacterial properties,^8^ is almost inert,^7^ and breaks down at pH conditions above 11.^9^ These properties have led to the use of PDA as a film or coating for obtaining safe biomedical implants and antibacterial substrates.^8^ Importantly, numerous studies demonstrated that PDA is biocompatible and biodegradable like natural melanin, with which shows similarities in structure and properties.^7^ This guarantees its safety and applicability for biomedical purposes.

### 2.3 PDA in Biomedical Applications

**Figure 3:**
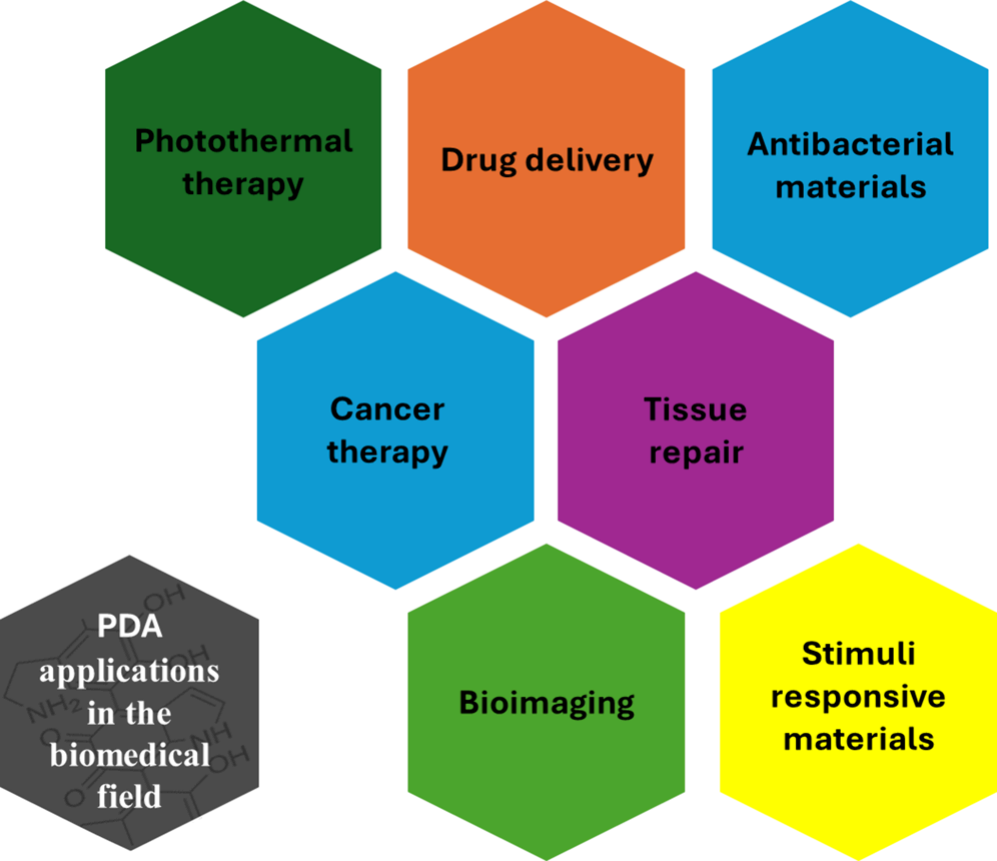
Main applications of PDA in the biomedical field.

PDA has been largely used for biomedical applications (Figure 3), either in the form of (nano/micro)particles or as films or coatings to obtain hybrid platforms with improved performances and functionalities.^10^ The different types of PDA arrangements that have been proposed depend on the particular applications imagined for this polymer, for example, preparation of surfaces with antifouling performance or nanocarriers for drug delivery applications. The properties of PDA previously illustrated (Section 2.2) manifest themselves similarly in the biological environment regardless of the type of PDA arrangement, since the interaction with biological fluids occurs at the interface with the PDA. A recent study, however, revealed that hemolytic activity and cellular toxicity depends also on PDA thickness, although the reasons for this behavior remain to be investigated.^11^

It was demonstrated that PDA coating can help to increase the biocompatibility of both organic/inorganic implants and nanosystems.^8,10^ PDA coating on implants, depending on the specific conditions, was useful to promote osteogenesis and osseointegration, to enhance cellular adhesion and proliferation, and to prevent bacterial infections and inflammation phenomena.

PDA has been often exploited to entrap and deliver a huge variety of drugs through covalent, π–π stacking, or electrostatic interactions. Thanks to the adhesive properties of the PDA polymer, surface coatings can be easily obtained on disparate materials for surface-mediated drug delivery applications.^3^ More commonly, PDA has been synthesized in the form of NPs or NP coatings to obtain efficient and colloidally stabilized nanocarriers (NCs) for drug delivery applications.^10,11^ Ho and collaborators prepared PDA-only NCs entrapping the anticancer camptothecin,^12^ while Nie et al. achieved a double delivery of chemotherapeutic agents by exploiting PDA coating to bind bortezomib on the surface of cholic acid-poly(lactide-*co*-glycolide) docetaxel-loaded NPs.^13^

As cited above, this polymer can be easily enriched with tumor-targeting agents containing a thiol or amine group. Thus, NCs were functionalized for the desired purpose with folate, glucosyl functional ligands, arginine–glycine–aspartate, and much more.^14^

Moreover, PDA-coated NCs have been exploited to prevent the early release of cargo and to obtain pH- and near-infrared (NIR) irradiation stimuli-responsive systems.^15^ It was observed that acidic pH boosts the release of hydrophilic and hydrophobic cargoes from PDA-based NCs compared with neutral-alkaline conditions. This behavior appeared to be particularly useful for chemotherapy application since healthy tissues and blood are characterized by pH conditions around 7.4, while tumor microenvironments usually have lower pH values around 5.0–6.0. In addition, the release of cargo stimulated by NIR radiation^15^ was efficiently adopted to promote the local release of payloads in the irradiated areas of the body. PDA, in fact, displays enhanced photothermal conversion in the NIR region of the spectrum and can convert the acquired energy into heat. The local heat generated upon NIR irradiation is able not only to stimulate the release of its drug payload but also to damage adjacent cells and tissue. Thus, PDA can serve for the photothermal ablation of the tumor. With this aim, Thirumurugan and co-workers enriched copper(II) benzene-1,3,5-tricarboxylate nanowires MRI contrast agent with a PDA coating,^16^ and combined the possibility of carrying out bioimaging and therapy simultaneously, while Li and collaborators combined photothermal and immunotherapy by designing a core–shell system consisting of CpG oligodeoxynucleotides-loaded PDA nanoplatforms decorated with hyaluronic acid.^17^

PDA has been further exploited for bioimaging purposes. Gallas et al. observed that the polymer can emit in the visible region of the spectrum upon excitation in the range 340–400 nm,^18^ while it has been extensively reported the possibility of obtaining fluorescent PDA-based probes upon treatment with H_2_O_2_ or other reactants without compromising their biocompatibility.^2^

Finally, Poinard and co-workers demonstrated that PDA possesses mucus-penetrating properties comparable with those of PEG, as well as enhanced cellular uptake,^19^ thus suggesting that PDA may represent a useful coating to implement in drug delivery systems directed to the intestine and lungs.

## 3 PDA and Liposomes: Ameliorative Effects of PDA Coatings

Liposomes are lipid-based vesicles consisting of one or more phospholipid bilayers that enclose an aqueous core.^20^ Commonly, phosphatidylcholine, phosphatidylserine, and sphingomyelin are used as the main constituents of the liposomal architecture, while cholesterol is included to tune the fluidity of the bilayer. Synthetic (phospho)lipids can also be included in the formulation of liposomes to obtain particular properties, and negatively or positively charged lipids can be embedded to adjust the surface charge of the vesicles. Liposomes have been extensively proposed as NCs for the delivery of bioactive agents or probes throughout the organism^21^ since they exhibit unique features that make them particularly interesting in this field. Indeed, their intrinsic core–shell structure enables the encapsulation of both hydrophobic and hydrophilic cargoes, individually or in combination, while their structure and composition mimic those of cell membranes, which make them generally biocompatible and able to facilitate the diffusion of their payload across the cell membrane. Notably, liposomes are rather simple to prepare and customize in terms of composition, size, and degree of lamellarity and can be easily surface-modified to improve their performance according to the specific purpose. However, major issues associated with the use of liposomes in the biomedical field include their limited colloidal stability over time, a generally uncontrolled release, and the need to introduce custom synthesized phospholipids into the bilayer to implement active targeting functionalities. In addition, once introduced into biological fluids, plasma proteins adsorb on their surface, thereby triggering immune responses and reducing their *in vivo* circulation lifetime.^11^ In this field, coating liposomes with polymers appears to be an attractive approach to control the release of cargo, to allow subsequent surface modification independent of the lipids used in the preparation, and to increase the stability of liposomes, both from a colloidal standpoint and with regard to interactions with biological elements *in vivo*. In this review, the advantages arising from the enrichment of the liposomes with PDA, as well as the method of preparation of these hybrid systems, will be presented (Table 1).

**Table 1:** Configurations and Main Applications of Hybrid Liposomes/PDA Systems and PDA Roles

configuration	application	PDA role	ref
liposomes embedded into PDA films	surface-mediated drug delivery	improved cellular interaction	(23, 24)
liposome@PDA	drug delivery (systemic administration)	improved cellular uptake	(25)
		easy functionalization	(14, 20, 26)
		modulation of the cargo release	(14, 27, 28)
		stimulus-responsive properties	(28−30)
		stabilization in biological fluids/stealth effect	(11)
	drug delivery (gastrointestinal and pulmonary administration)	mucus-penetrating properties	(31)
	photothermal therapy	photothermal effect	(28)

In detail, liposomes have been combined with PDA for two main purposes: (1) to simultaneously exploit the delivery capabilities of the liposomes and the outstanding properties of PDA (see Sections 2.2 and 2.3) or (2) to use the vesicles as mere templating agents to obtain uniform, well-dispersed PDA-based nanostructured materials in a very simple way. The latter case is well represented by the work of Awasthi et al., who exploited liposomes as a starting material to fabricate a uniform nanostructured PDA coating on a substrate instead of random polymer aggregates with remarkable antifouling performance against *S. aureus* and *E. coli*.^22^ The first case, instead, is the most interesting for its application possibilities in the field of life sciences and is embodied by numerous works that will be presented in the next paragraphs on a case-by-case basis focusing on the ameliorative effects conferred by enriching the liposomes with PDA.

### 3.1 Improved Cellular Interaction

**Figure 4:**
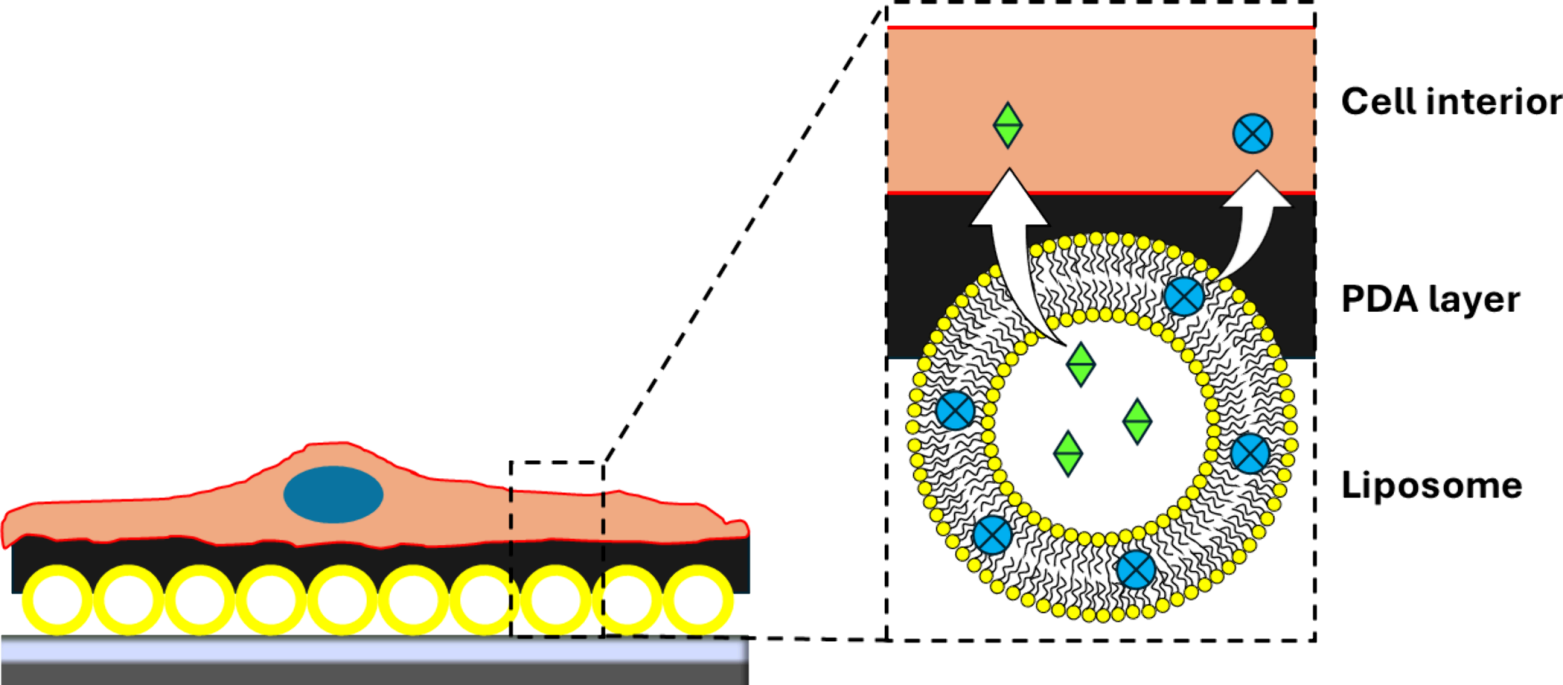
Illustration of the use of PDA as a coating for supported liposome layers. The coatings thus obtained have improved interactions with cells and are useful as surface coatings in surface-mediated drug delivery applications.

Layer of liposomes can be embedded in a PDA matrix to obtain biocompatible surfaces with improved cellular interaction (Figure 4). For example, liposomes were coated with a PDA matrix to provide an adhesive substrate for myoblast cells for an efficient surface-mediated drug delivery.^23,24^ In detail, liposomes loaded with a fluorescent cargo were prepared and coated with a layer of PDA, and then the composite system was exposed to myoblast cells. A fluorescent signal corresponding to the loading, which depended on the cell residence time and thickness of the PDA layer, was observed in the cells. The signal rose up to a certain PDA thickness corresponding to 30 min of polymerization and then started to decrease. Also, the fluorescence signal decreased with increased cell adhesion time.^24^ The same group also assessed the interaction of PDA-coated liposomes with myoblast cells.^25^ They observed that the PDA coating preserved the biocompatibility of the liposomes and might improve their cellular uptake.

### 3.2 Easy Functionalization

**Figure 5:**
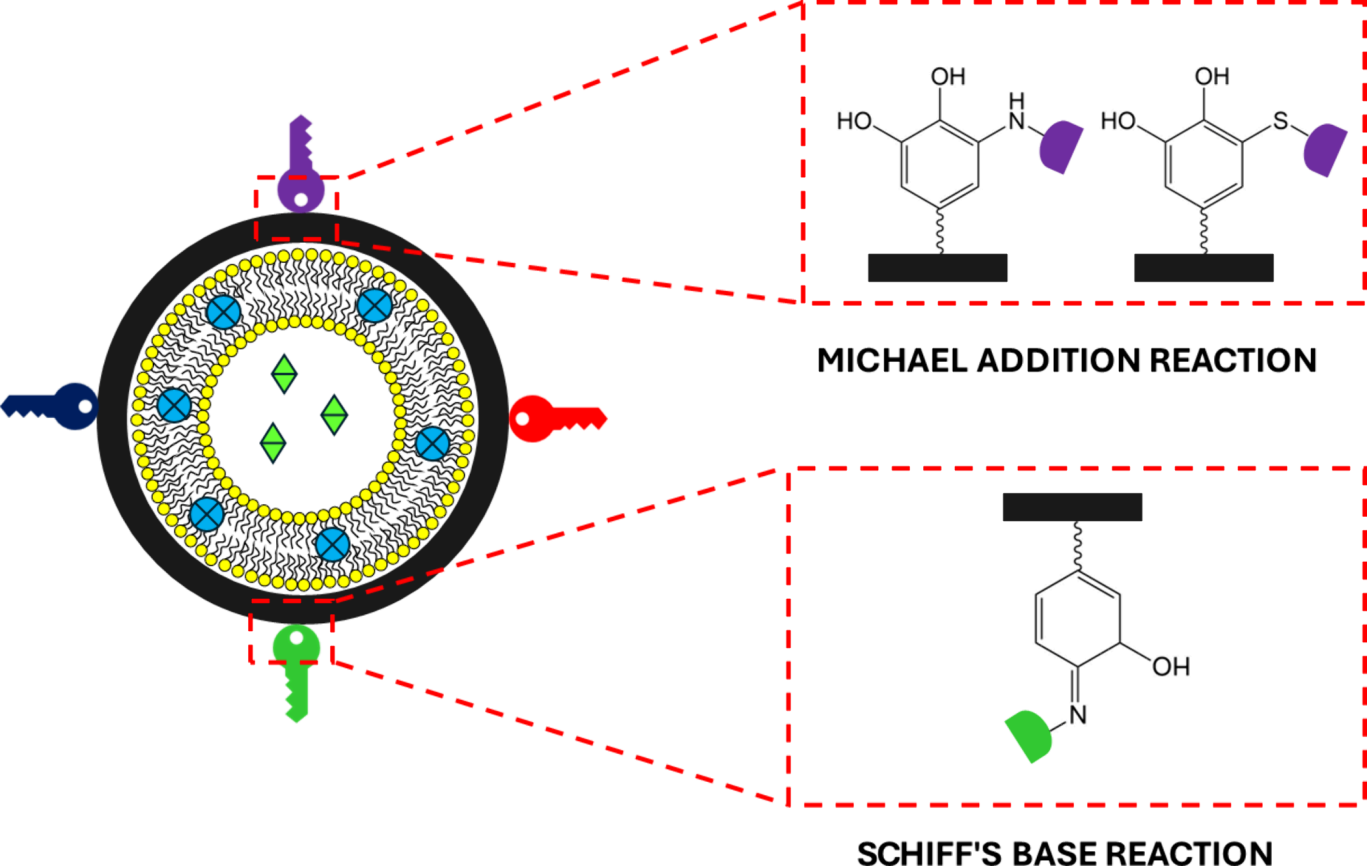
Sketch depicting the possibility of easily functionalizing PDA coatings with amines and thiols via Michael addition or Schiff’s base reaction.

**Figure 6:**
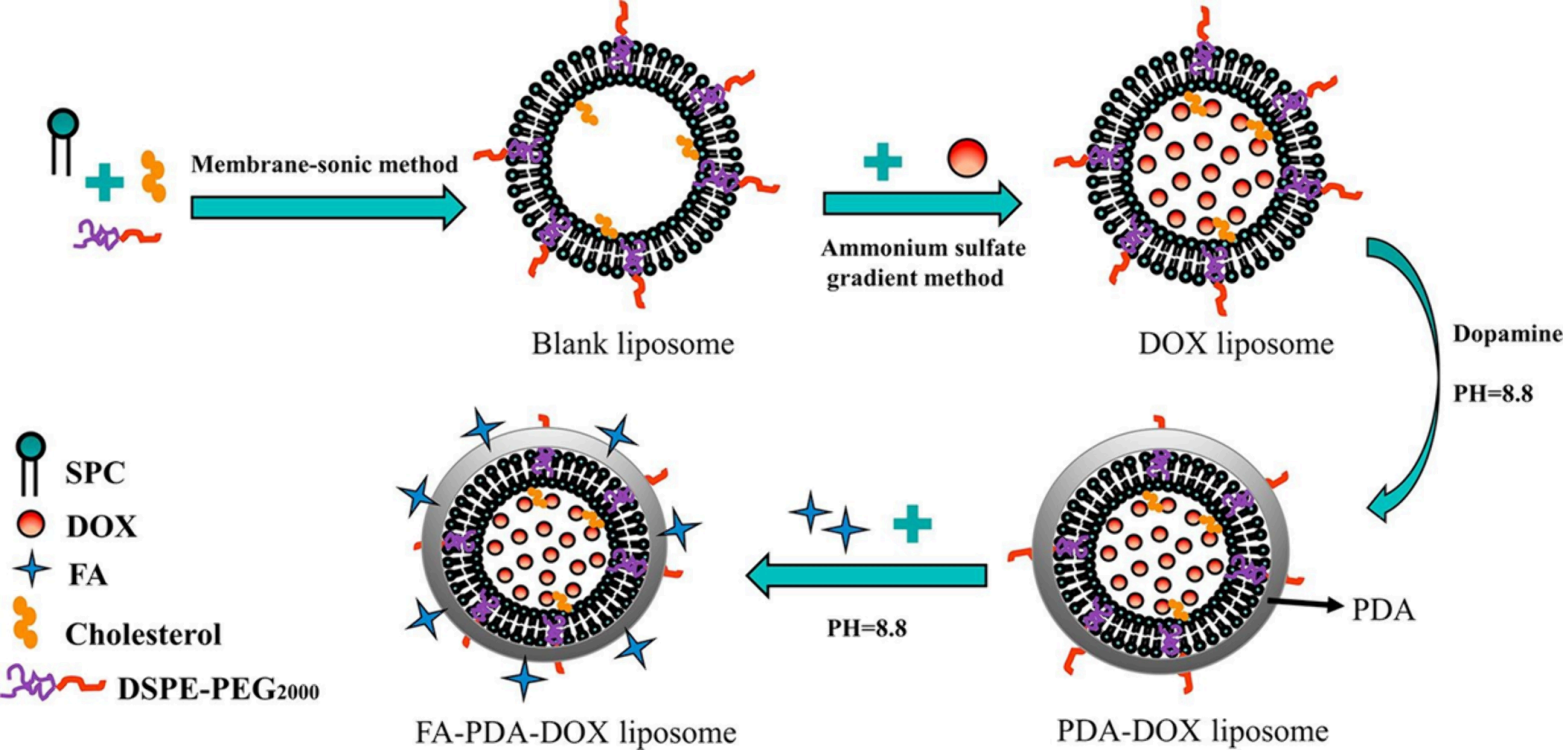
Schematic illustration of the preparation of DOX-loaded liposomes coated with PDA and further functionalization with folic acid. Reprinted with permission from ref (20). Copyright 2019 Elsevier.

Considering the most common configuration, i.e., liposomes individually coated with a shell of PDA, the ability of PDA to form covalent bonds with substrates having thiol or amine groups under mild conditions has been exploited to functionalize liposomes in a simple manner without the need to purchase and use phospholipids specifically modified with the desired functional group. Specifically, the PDA shell can bind thiol or amine groups via Michael addition reaction and amines also via Schiff’s base reaction (Figure 5). This has resulted in being particularly useful for cancer therapy applications to achieve a targeted release toward the tumor and minimize the side effects toward healthy tissues. To this aim, PDA-coated liposomes were functionalized with folic acid by exploiting its amine functionality, incubating the coated vesicles with folic acid in tris(hydroxymethyl)aminomethane (TRIS) buffer at pH 8.5, and leaving it to react for half an hour at room temperature (Figure 6).^20^ Following the same approach, a PDA shell was exploited to introduce chains of PEG onto the surface of the liposomes without using a PEG-modified lipid.^26^ PEG is essential to stabilize and increase the circulation time of systemically administered nanoparticles. PEGylation represents a widely adopted approach to achieve stealth systems; therefore, implementing it using PDA could be a more accessible and universal strategy.

### 3.3 Modulation or Extension of Cargo Release Capacity

**Figure 7:**
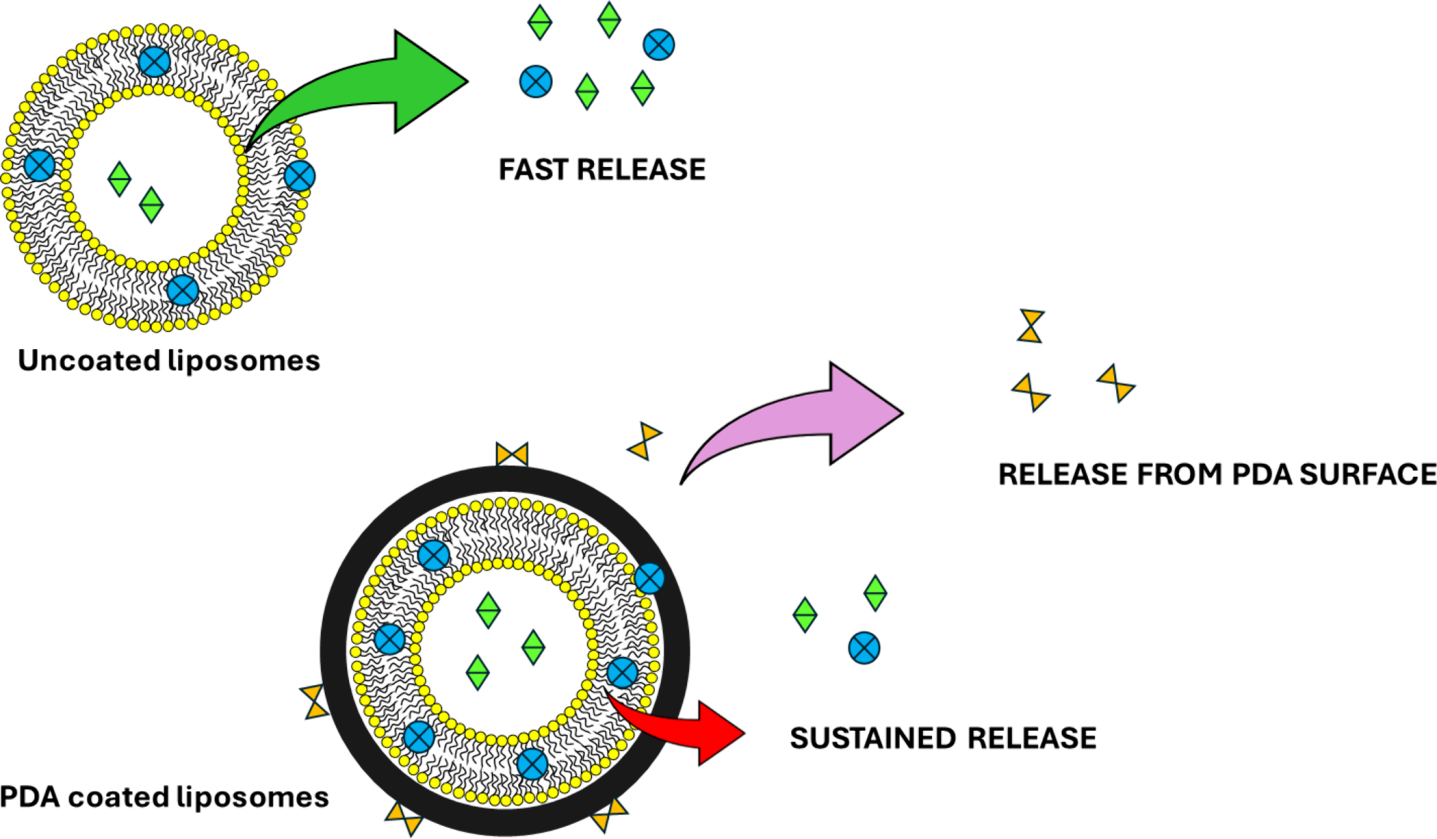
Illustration of the ability of the PDA coating to modulate the release of the cargoes embedded into the liposomes, as well as to entrap and release compounds from the PDA surface.

The presence of a PDA shell around the vesicles resulted in an effective strategy to modulate the kinetic release of the bioactive cargo loaded into the liposomes and prevent its premature release before reaching the site of interest (Figure 7). Lim and co-workers compared the release of the acetaminophen from naked and PDA-coated liposomes and observed that the polymer-coated samples exhibited a significantly decreased release rate, probably because the PDA acts as a barrier to the diffusion and also because of the π–π interactions that the polymer may establish with the drug.^27^ Moreover, the PDA shell provides an additional storage site for bioactive agents on the vesicle surface. For example, methylene blue has been adsorbed and delivered by PDA-coated liposomes for photodynamic therapy applications.^32^ In this case, the adsorption phenomenon was mostly due to electrostatic interactions between the negatively charged PDA shell and the cationic photosensitizer.

### 3.4 Obtainment of Stimulus-Responsive Carriers

**Figure 8:**
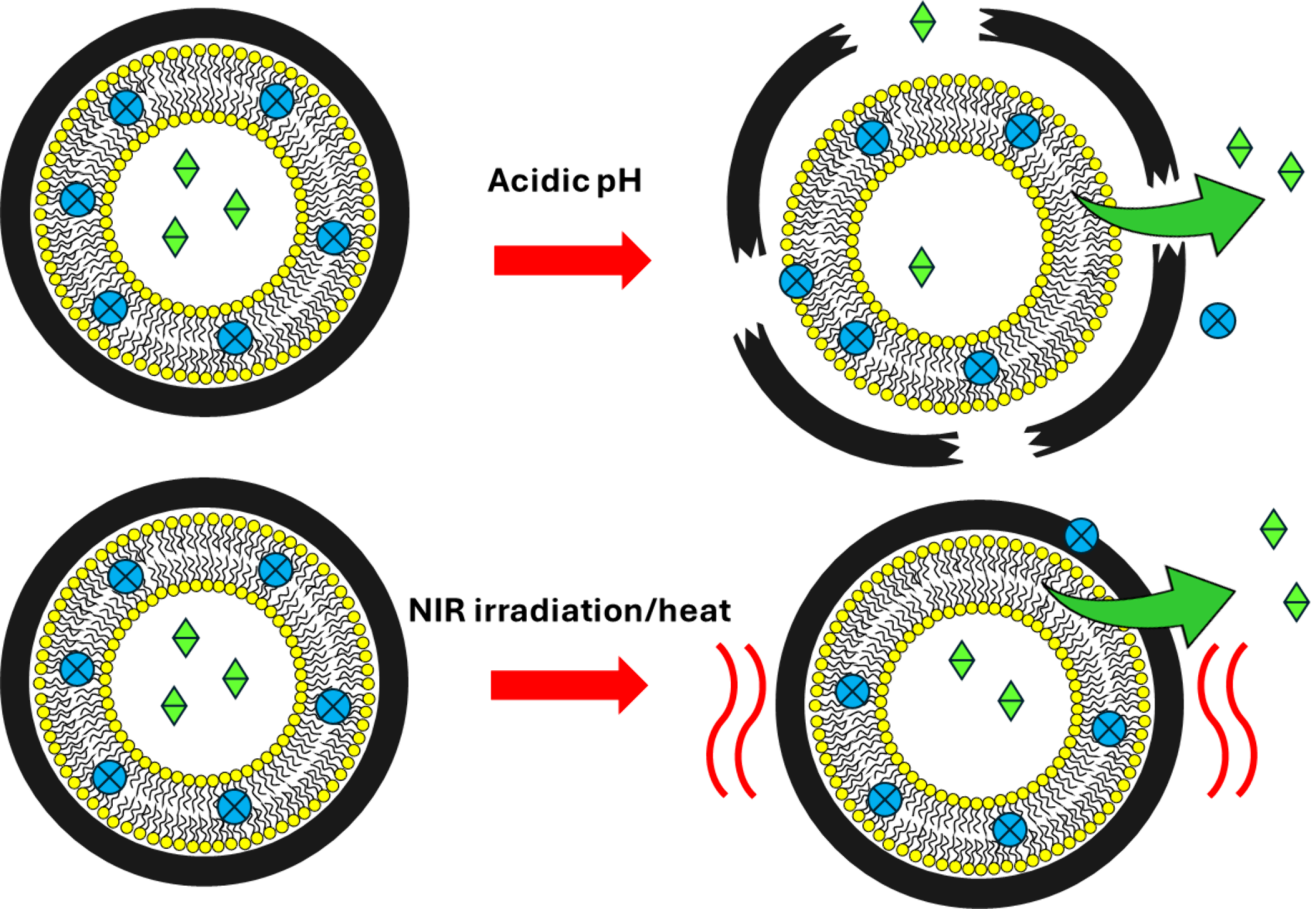
Sketch illustrating the possibility of exploiting the PDA coating around liposomes to obtain pH- or light-stimulus-responsive systems.

Besides decelerating the release kinetics of the cargoes, the PDA coating was effectively exploited to obtain stimulus-responsive systems and achieve a controlled release (Figure 8). Specifically, to obtain temperature-responsive carriers, liposomes have been equipped with mixed coatings composed of PDA and thermoresponsive poly(*N*-isopropylacrylamides) (pNiPAAm) polymers, which are realized by copolymerizing DA and the specific kind of pNiPAAm under consideration in liposomal suspension.^29^ Instead, Zong et al. prepared 5-fluoruroacil-loaded liposomes and coated them with PDA to achieve a controlled pH-dependent release of the drug. They found that the release resulted was enhanced in acidic pH conditions,^30^ which is typical of the tumor microenvironment. This behavior has been ascribed to a possible decomposition of the polymeric layer in acidic conditions, but this topic is still under debate. Finally, the release of drugs from PDA-coated liposomes was stimulated by NIR radiation, likely due to the heat generated by the photothermal effect.^28^

### 3.5 PDA for Mucus-Penetrating NCs

**Figure 9:**
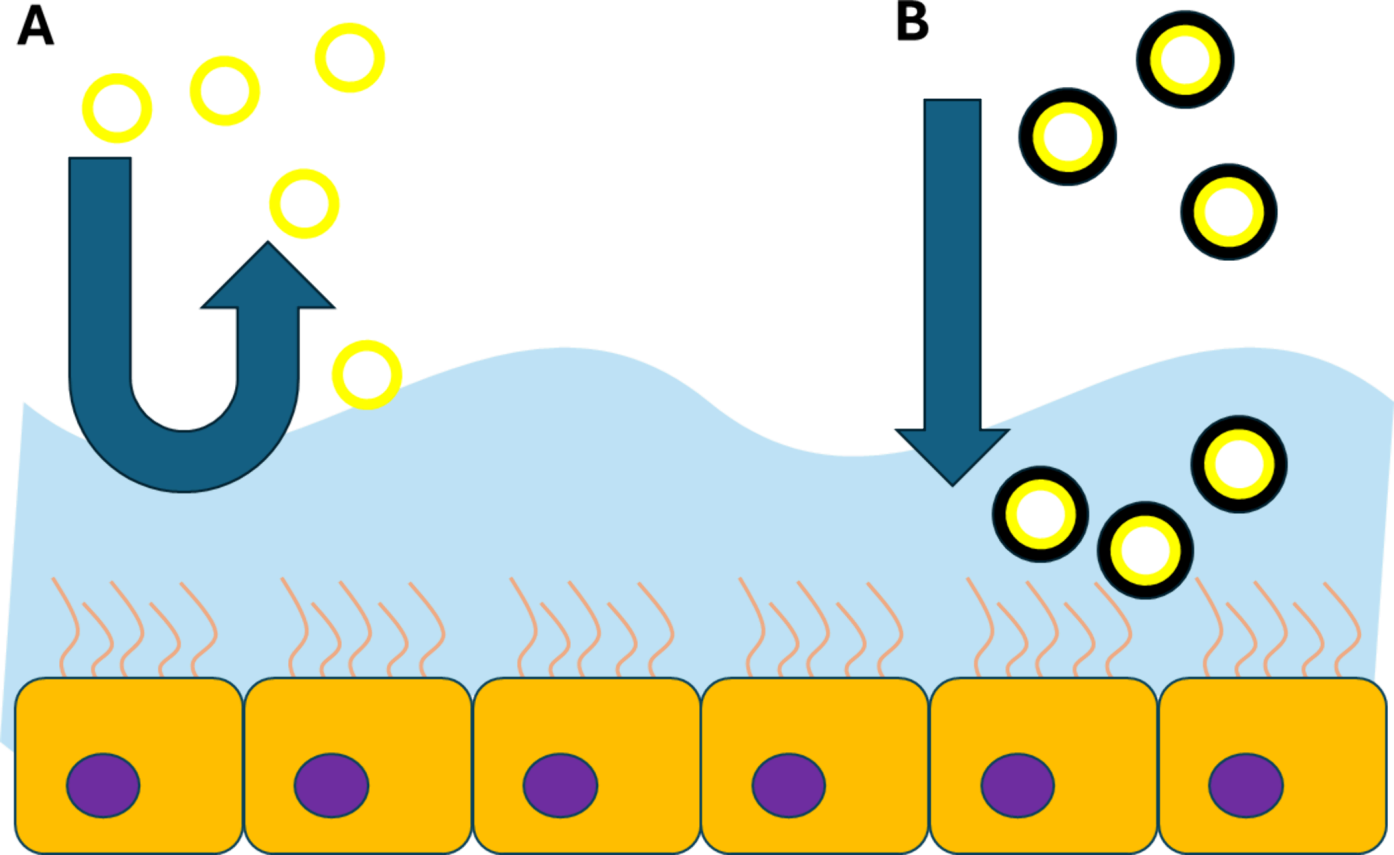
Picture showing the mucopenetrating behavior of the liposomes. Conventional liposomes (A) usually unable to cross the mucus layer, whereas PDA-coated liposomes (B) succeed in reaching the underlying layers.

**Figure 10:**
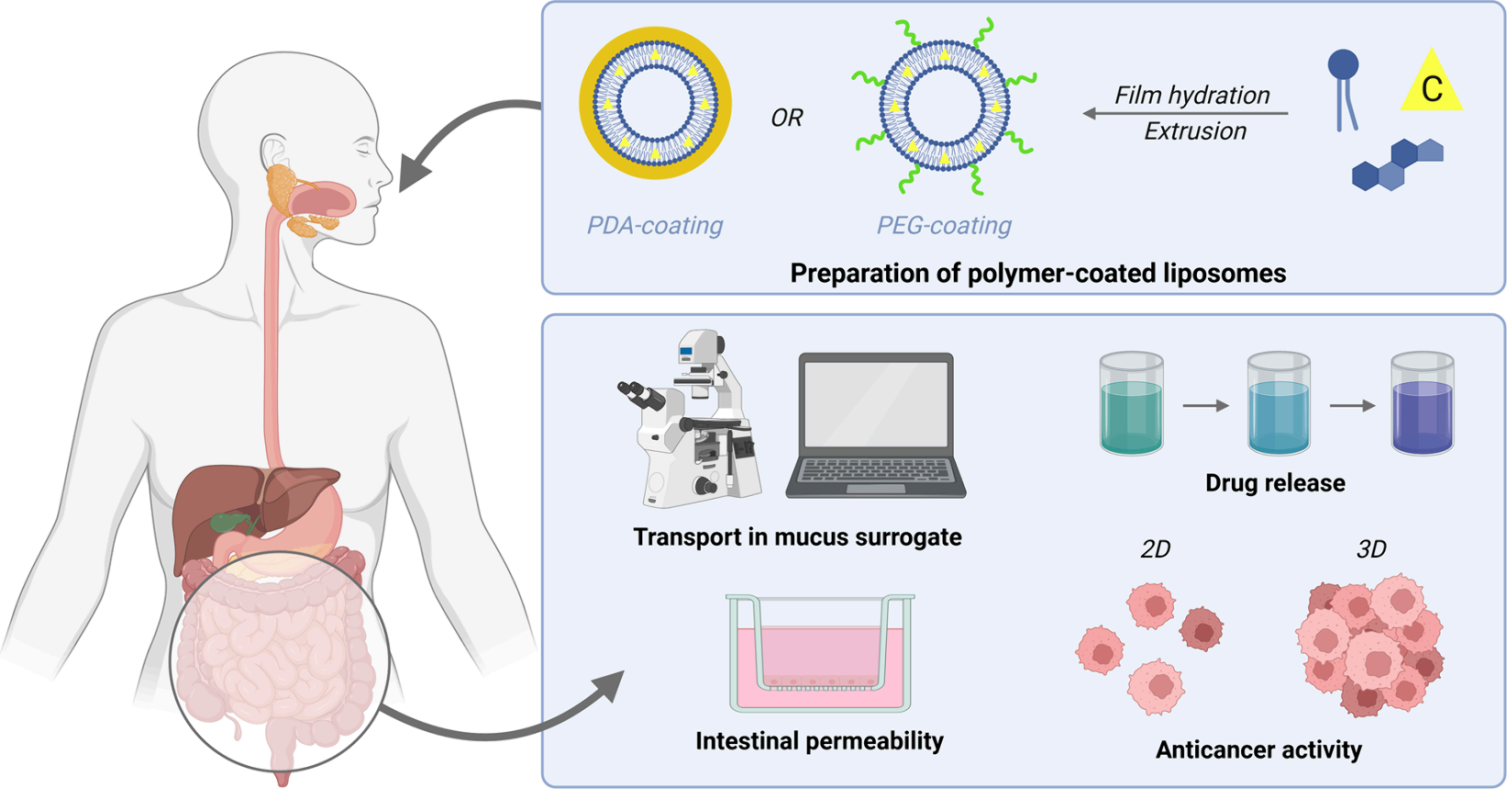
Illustration of the implementation of PEG or PDA coatings to liposomes as a strategy to improve their efficacy in the oral administration of anticancer drugs. Reproduced from ref (31) under Creative Commons license.

PDA was successfully tested as a coating of liposomes intended for oral administration of a chemotherapeutic agent to improve their performance at the intestinal level and confer them mucopenetrating properties (Figure 9).^31^ Multiple particle tracking analysis experiments have suggested that PDA-coated liposomes exhibit subdiffusive behavior in a mucus surrogate with diffusivity values comparable with or even better than those of PEG-coated liposomes. In addition, *in vitro* intestinal permeability experiments performed on models containing mucus-producing cells showed a better permeability of PDA-coated liposomes compared with PEGylated ones. Thus, these results confirmed previous observations on PDA- and PEG-coated polystyrene NPs^19^ and suggest that the implementation of PDA coatings may be a useful strategy to improve the efficacy of orally administered NPs (Figure 10).

### 3.6 PDA as Stealth Coating

**Figure 11:**
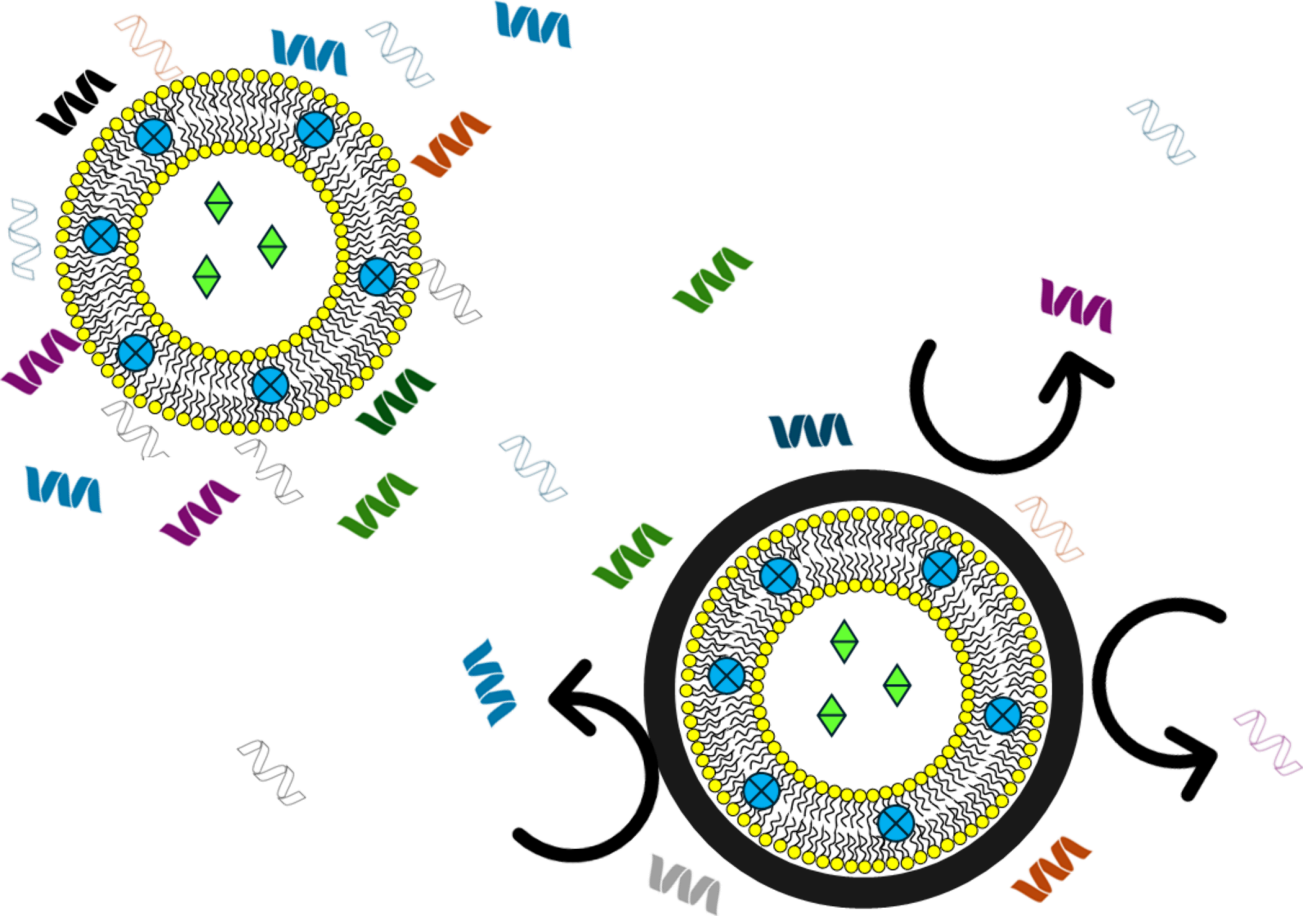
Picture showing the ability of the PDA coating to generate stealth liposomes by reducing/modulating the adsorption of proteins onto their surface once they are in contact with biological fluids.

**Figure 12:**
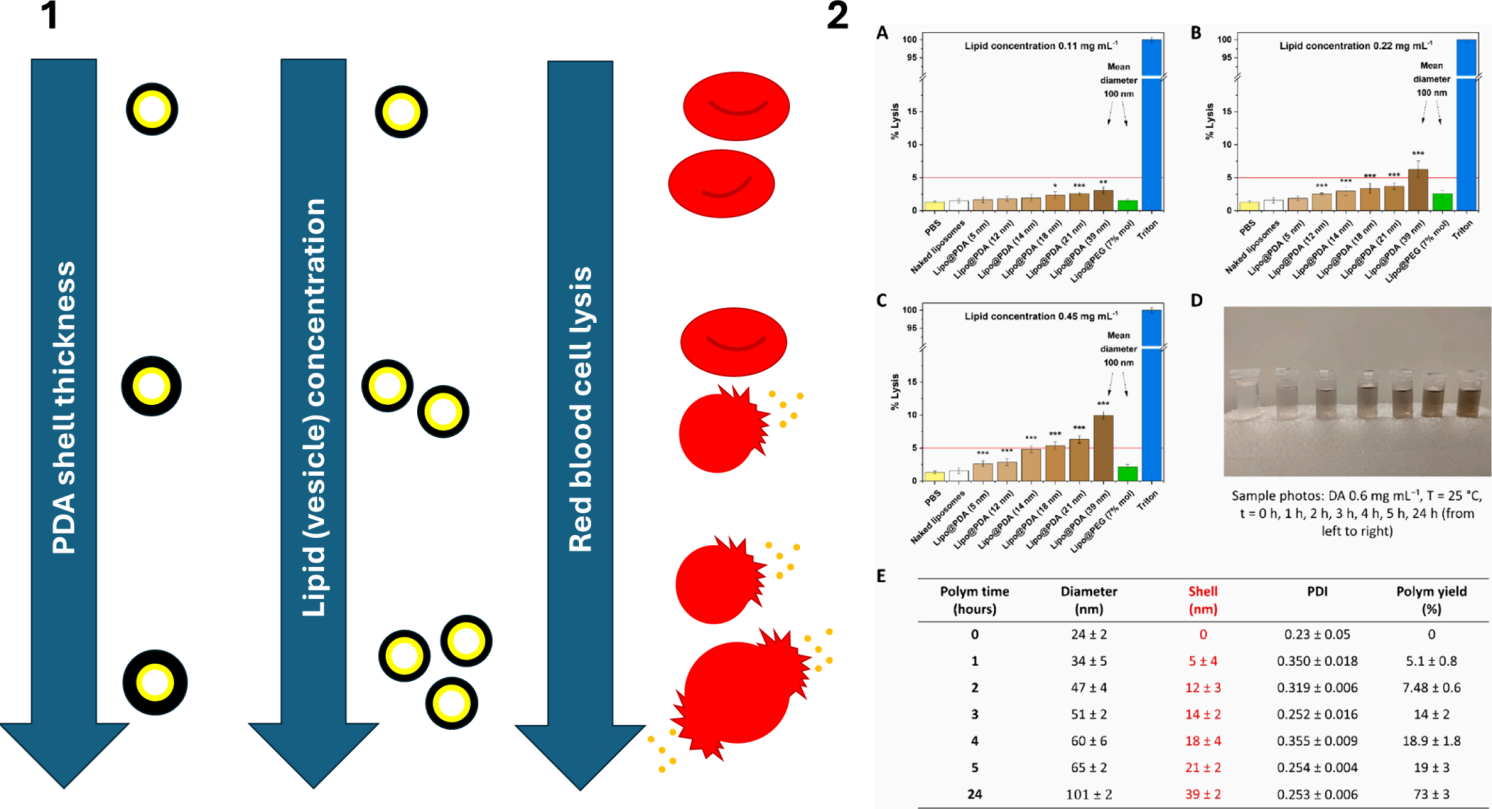
Panel 1: Dependence of the extent of lysis induced in red blood cells following incubation with liposome (Lipo)@PDA on the PDA shell thickness and vesicle concentration. Panel 2: (A–C) Percentage of red blood cell lysis induced by PEG- and PDA-coated liposomes (Lipo@PEG and Lipo@PDA, respectively) at different final lipid concentrations and increasing PDA shell thickness; (D) photographs of PDA-coated liposomes obtained at increasing polymerization time; and (E) physicochemical characterization of PDA-coated liposomes in terms of diameter, polymer coating thickness, polydispersity index, and polymerization reaction yield (%). Panel 2 reprinted with permission from ref (11). Copyright 2024 Elsevier.

To ensure the stability of liposomes in a biological environment and make them long-circulating or stealth, it is necessary to coat the lipid vesicles with hydrophilic polymers, such as PEG, to reduce opsonization and phagocytosis. PDA-based coatings appear to be useful for the same purpose as they would be able to interact favorably with serum proteins, thereby leading to the formation of the so-called protein corona similar to that of conventional stealth polymers (Figure 11). In a recent work, the use of PDA as a coating for liposomes was proposed and tested in order to increase their stability in biological fluids and obtain stealth systems for drug delivery applications.^11^ The authors thoroughly investigated the procedure of preparing PDA-coated liposomes and studied: (i) their colloidal stability, (ii) their protein corona after incubation with fetal bovine serum, (iii) their haemolytic behaviour towards red blood cells and (iv) their cytotoxicity towards human lung cells. The results were compared with those obtained using a PEG-based coating, which represents the current standard strategy for obtaining liposomes that are safe and invisible to the immune system. A strong similarity from a qualitative and quantitative standpoint was observed between the protein coronas formed around the two polymer-enriched liposomes, thereby suggesting that coating liposomes with PDA may be a useful strategy to obtain stealth particles without using PEG, whose massive use is now of growing concern because of the increase in adverse reactions reported in the population after the administration of PEGylated formulations. Interestingly, it was observed there was a dependence for the hemolytic response on the PDA-coating thickness (as well as lipid concentration) with thicker coatings inducing higher percentages of lysis. Thus, the authors highlighted the necessity of taking into account this parameter when designing PDA composite architectures to be administered *in vivo* (Figure 12). Also, negligible cytotoxic effects were observed after incubating the above-cited kinds of vesicles with H441 human respiratory cells.

### 3.7 Obtainment of Multifunctional Nanocarriers

**Figure 13:**
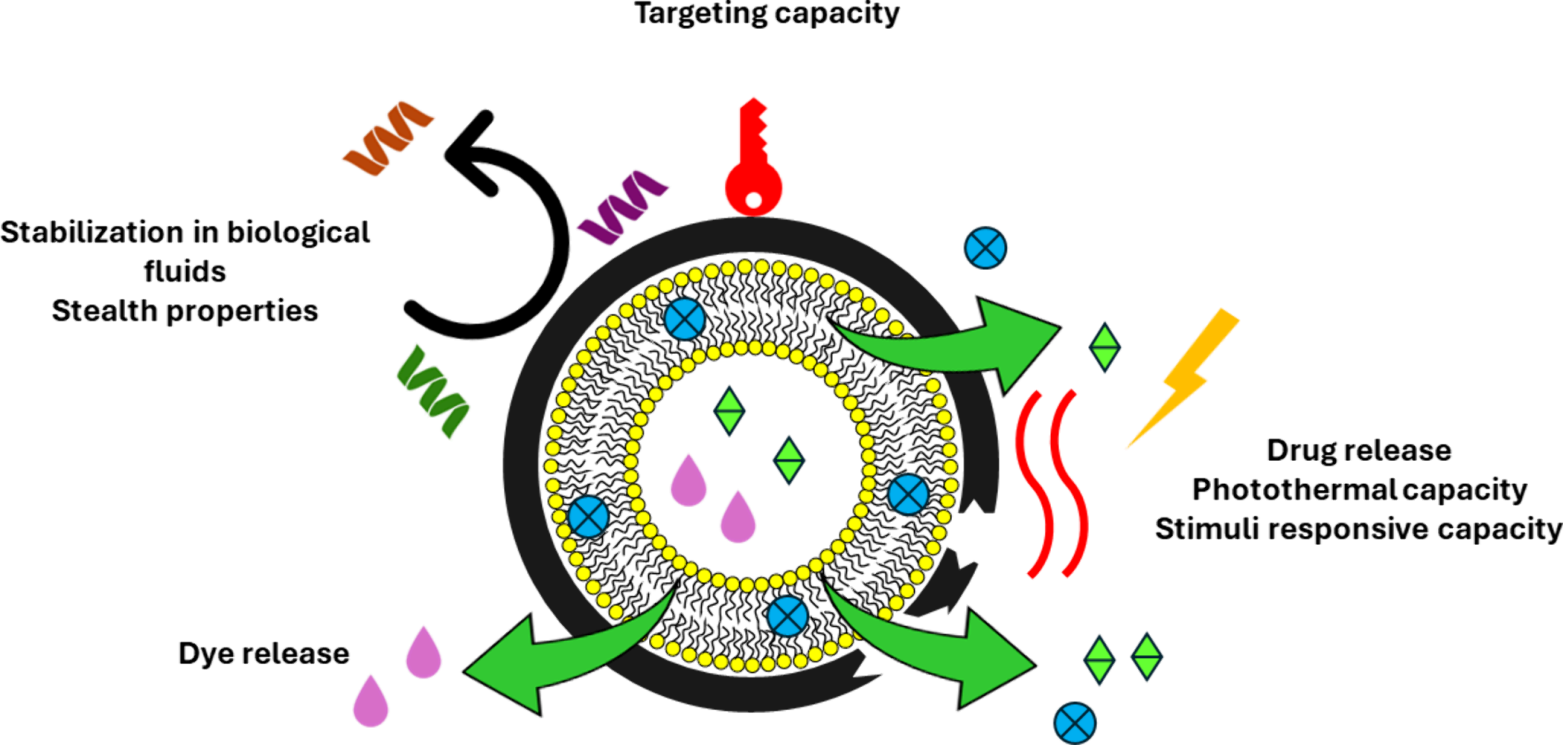
Use of the PDA coating to obtain multifunctional nanocarriers by simultaneously exploiting its properties.

The manifold properties of the PDA (Figure 13) were exploited by Lu et al. to obtain a multifunctional liposome-based nanocarrier.^28^ Specifically, liposomes loaded with doxorubicin (DOX), an anticancer drug, and indocyanine green (ICG), a fluorescent dye useful both for imaging purposes and as a photosensitizer (PS) and photothermal agent, were designed and coated with PDA for the treatment of breast cancer. The presence of the polymer prevented premature drug release and enhanced the photothermal effect under 808 nm laser irradiation. Indeed, under these conditions, DOX release was increased, especially under acidic pH conditions, thus confirming the ability of PDA to make the carrier responsive to external stimuli. In synergy with the chemotherapeutic effect of the DOX, the photothermal effect of PDA and the photodynamic/photothermal effect of the ICG were also actively exploited to induce tumor cell death more effectively via the increase in local temperature resulting from irradiation. In fact, upon NIR irradiation, photothermal agents emit heat, which can result in the thermal ablation of the tumor, while PSs produce ROS. Once coupled, the generated heat may improve the photodynamic action of the PS by producing ROS and, additionally, the generated ROS make cells more sensitive to the photothermal action. Further, Honmane and collaborators exploited the coating of PDA from multiple perspectives. It resulted in being useful both for easily introducing the folic acid on the surface of the liposomes for a targeted tumoral uptake and also for modulating the release of the cargo. In fact, the presence of the PDA coating slowed the release of the cargo at neutral alkaline conditions and sped it up in acidic conditions characteristic of the tumor environment.^14^

## 4 PDA-Modified Liposomes: Preparation

Strategies for the preparation of naked liposomes will be not discussed in this review as this topic is already well covered by other recent works.^21^ Conversely, the association of liposomes with PDA will be illustrated by distinguishing between two main configurations: liposomes embedded into PDA films and the coating of individual liposomes with PDA shells (Lipo@PDA).

### 4.1 Liposomes Embedded into PDA Films

**Figure 14:**
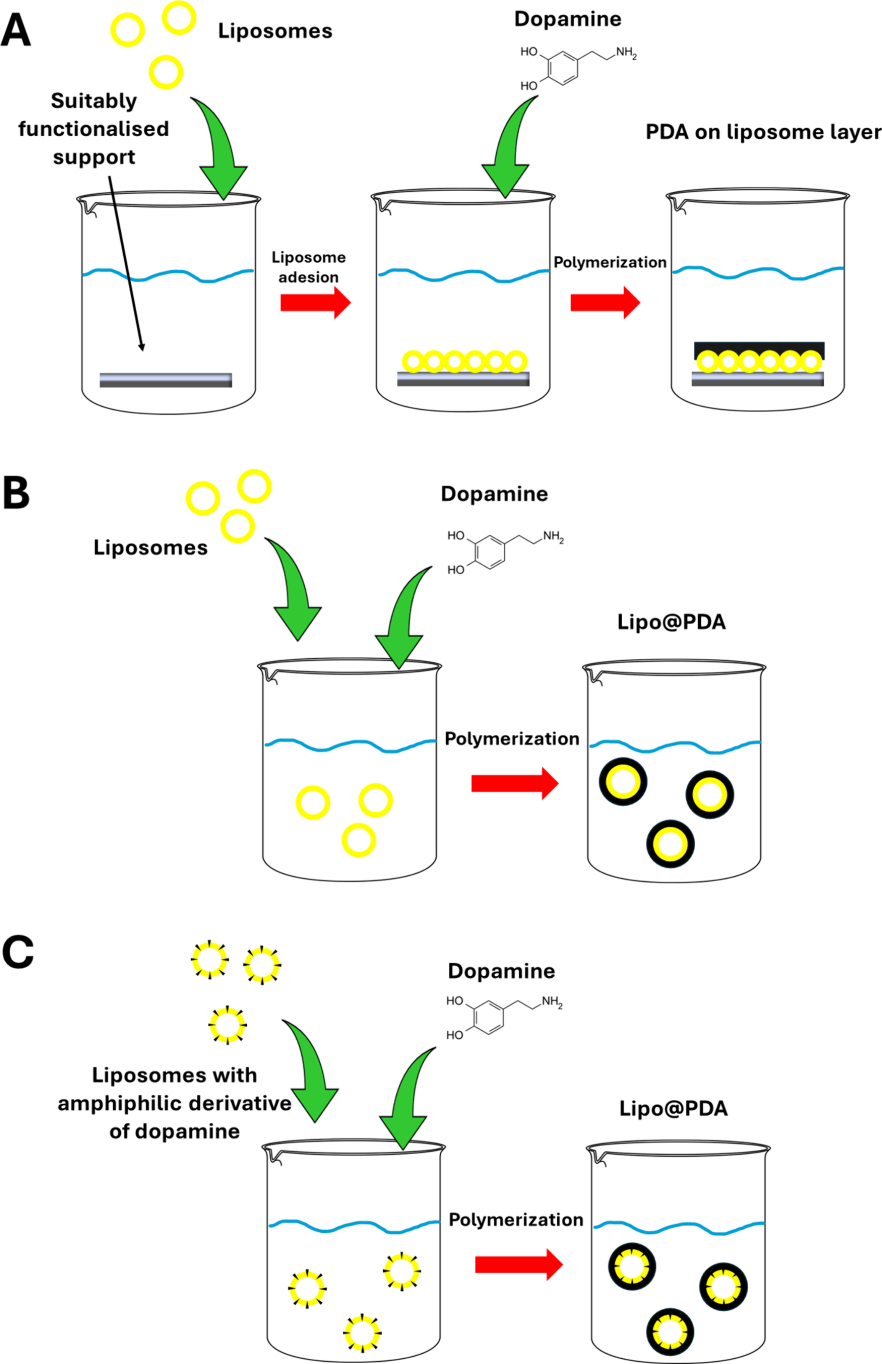
Preparation strategies for hybrid liposome/PDA systems: (A) platforms consisting of a liposome layer adhered to a suitably functionalized support and subsequently coated with PDA by *in situ* polymerization of DA; Lipo@PDA obtained via *in situ* polymerization of DA (B) in a liposomal suspension and (C) in the presence of liposomes featuring an amphiphilic dopamine derivative in the lipid bilayer.

**Figure 15:**
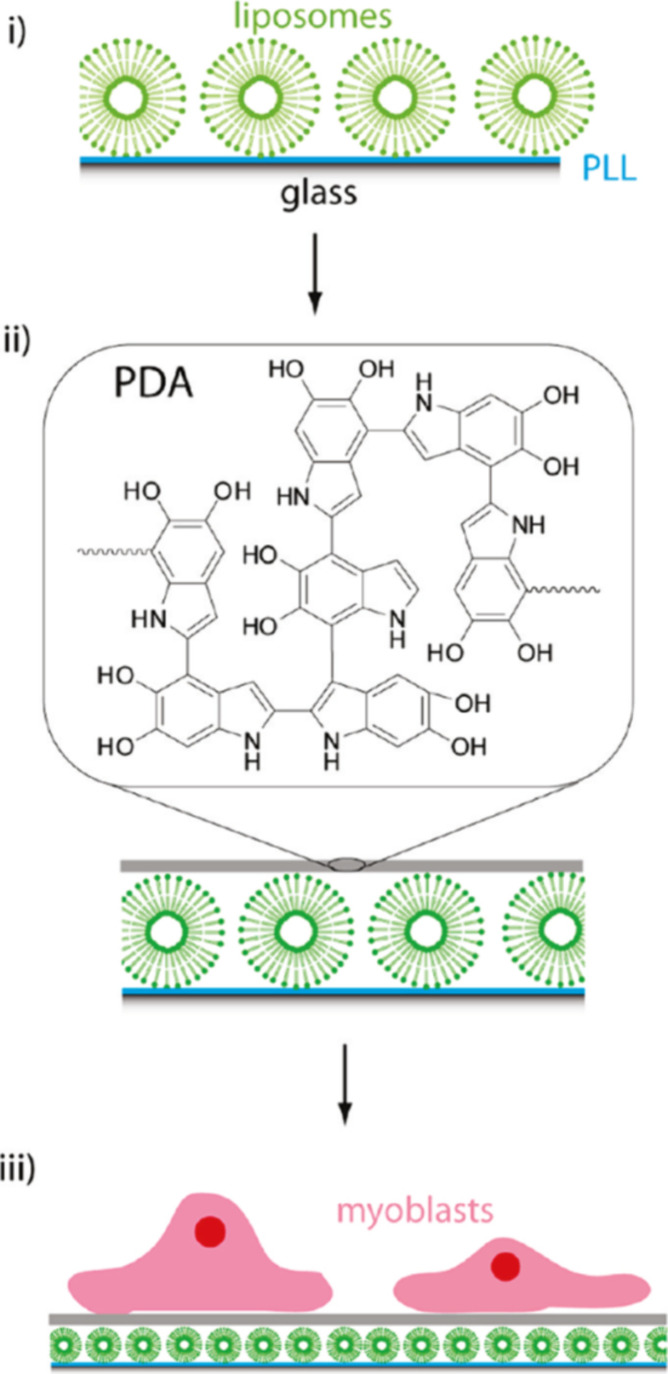
Illustration of the assembly of liposomes-adsorbed PLL-precoated substrates with a PDA layer following exposure to myoblast cells. Reprinted with permission from ref (24). Copyright 2011 American Chemical Society.

The first work reporting the assembly of liposomes with PDA was published in 2011 by Städler’s group.^24^ Here, platforms for surface-mediated drug delivery were prepared by coating a layer of liposomes adhered onto a poly(l-lysine) (PLL) substrate with PDA where liposomes were intended as a drug reserve. A general representation of the proposed synthetic strategy is provided in Figure 14A. In detail, Städler and collaborators exposed the substrate with anchored liposomes to a dopamine hydrochloride solution in TRIS buffer at pH 8.5 and replaced the solution every 30 min (Figure 15).^24^ The same group provided a composite coating onto PLL substrates with multiple liposome–PDA layers.^23^ In this case, different buffers, namely, borate, phosphate, and TRIS buffer, at pH 8.5 were tested. It was observed that the borate buffer did not lead to PDA formation, probably because of the covalent ester interaction between the boric acid and the DA vicinal diol. Instead, phosphate and TRIS buffers induced the formation of PDA in different amounts, thereby corroborating the theory that the TRIS molecule is integrated into the polymer structure.^23^

### 4.2 Lipo@PDA

The strategies proposed to obtain a PDA coating on the surface of isolated liposomes are quite simple and generally follow the same scheme. Commonly, the strong templating effect of liposomes is exploited by simply inducing the *in situ* polymerization of DA in a liposomal suspension in TRIS buffer at pH 8.5^14,20,26,28,30^ or phosphate buffer at pH 8.0 (Figure 14B).^5,11^ In fact, it has been observed that to ensure a good PDA coating, it is important that the anchoring takes place at an early stage of the polymerization process or, better, that the polymerization is performed in the presence of the vesicles to be coated. Indeed, when the PDA particles are already formed, the majority of uncyclized aminoethyl groups have already converted to indoles, which are less prone to adhesion.^1^ It should be noted that the type of buffer used to raise the pH is not indifferent from the polymerization process. Small-angle neutron scattering data suggested that two-dimensional structures form in phosphate buffers, while apparently three-dimensional fractal structures prevailed in the TRIS buffer. Researchers also suggested that PDA formed in TRIS buffer might contain (covalently) bonded TRIS molecules, especially at low DA concentration and at the initial phase of the PDA formation process.^4^

When the polymerization of PDA was obtained in the presence of liposomes, the massive deposition of the polymer on the internal walls of the reaction vessel was not observed, as occurs in the absence of vesicles.^5^ The formation of PDA-only particles also appeared limited, thereby indicating that polymer deposition occurs predominantly on the surface of the liposomes. In addition, it was demonstrated that polymerization does not occur within the aqueous core of the liposomes. For this purpose, sucrose-loaded Lipo@PDA vesicles were prepared and were subsequently dispersed into a glucose solution. The sucrose solution trapped within the vesicles possessed a higher density compared with glucose, which led to the settling of Lipo@PDA structures as a dark pellet within 1.5 h. The complete discoloration of the supernatant solution indicated the successful grafting of all PDA polymer onto the liposome surface and the preservation of the vesicles’ aqueous core during DA polymerization.^11^

**Figure 16:**
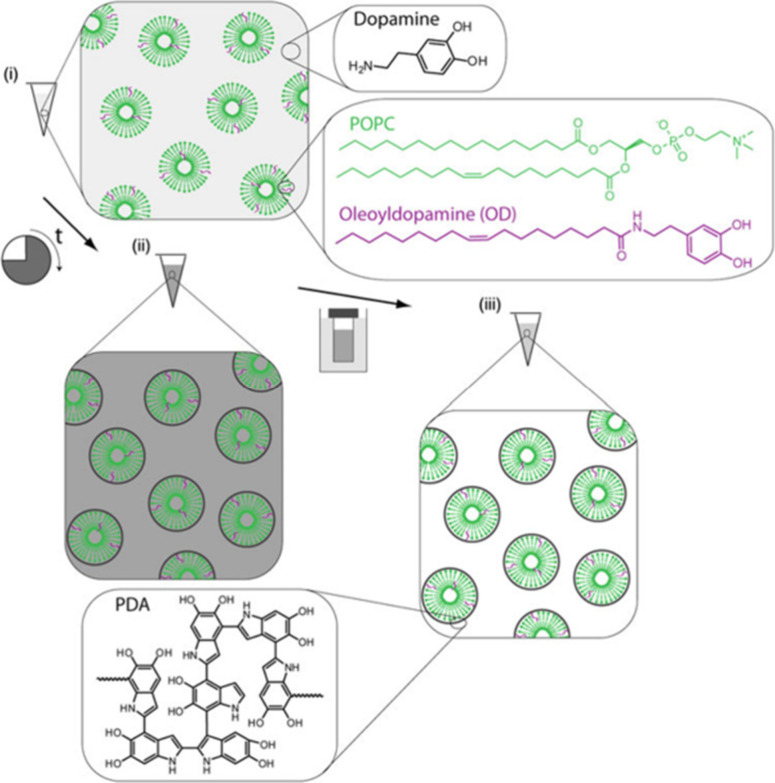
Sketch depicting the procedure for preparing Lipo@PDA starting from vesicles with OD in the bilayer. (i) A DA solution is mixed with the liposomes and (ii) allowed to react; then (iii) the sample is purified through dialysis. Reprinted from ref (25) under Creative Commons license.

Van der Westen and co-workers proposed a slightly different strategy for Lipo@PDA preparation by synthesizing oleoyldopamine (OD), an amphiphilic derivative of DA, with a hydrophobic anchor capable of intercalating into the lipid membrane of liposomes.^25^ OD was incorporated into the liposome membrane to aid in the grafting of PDA by serving as an anchor as it copolymerized with dopamine/PDA (Figure 14C and Figure 16). The researchers observed that greater amounts of OD resulted in accelerated PDA growth rates with identical DA concentrations. In contrast, increasing DA concentration had no impact on the growth rate of PDA; however, sample aggregation occurred more rapidly.

Whatever strategy is used, after the formation of the PDA shell around the vesicles has been achieved, the particles are generally purified by dialysis^31^ or through multiple washes and centrifugation in order to remove unreacted DA and unpolymerized molecular products.^26,30^ The impact of reaction conditions on the final PDA shell thickness and polymerization yield has also been explored. In general, increasing temperatures, initial DA concentration, and reaction times lead to thicker PDA coatings. Higher temperatures and longer times also result in higher polymerization yields, whereas the yield is not affected by the initial DA concentration.^11^ A further development involved the coating of zwitterionic liposomes with a mixture of PDA with nonionic polymers poly(*N*-vinylpyrrolidone) (PVP), poly(vinyl alcohol) (PVA), or PEG in order to understand whether the presence of the nonionic polymer affects the formation of PDA shell in terms of growth rate and colloidal stability.^33^ The synthesis was carried out by incubating vesicles with DA (1 mg/mL) in TRIS buffer at pH 8.5 in the presence of one of these polymers. It was observed that the addition of small amounts of PEG or PVA allowed the coating of liposomes without affecting their polydispersity, whereas increasing amounts of PEG and PVA led to the aggregation of the samples. However, the presence of PVP in the DA solution impaired the deposition of the polymer around the vesicles. Finally, capsosomes, i.e., liposomes embedded within a polymeric carrier capsule, were obtained through a multiple-step procedure proposed by Hosta-Rigau and collaborators.^34^ To this aim, a suspension of silica particles in TRIS buffer was first incubated with the polymer precursor layer PLL, washed in TRIS, and then resuspended in a liposome solution, washed again, and incubated in a poly(methacrylic acid)-*co*-(cholesteryl methacrylate) solution. If needed, a second liposome deposition step was carried out. The PDA shell was realized by incubating the obtained particles in a DA solution (8 mg/mL) in TRIS at pH 8.5 for 16 h. The final PDA-based capsosomes were obtained by dissolving the silica core particles using a 2 M hydrofluoric acid/8 M ammonium fluoride solution at pH 5.

## 5 Conclusions

In this mini-review, a brief glimpse into the properties and applications of the PDA in the biomedical field has been provided, while particular attention was paid to illustrating the properties and the synthetic and applicative aspects of PDA–liposomes composite architectures. It has emerged that combining the outstanding and versatile properties of PDA with the delivery abilities of liposomes can represent a powerful, affordable, and easy technique to obtain stable multifunctional (nano)systems featuring a targeted, modulated, or stimuli-responsive (NIR, pH) release. In addition, promising results have emerged on the ability of PDA coating to provide stealth and mucopenetrating liposomes without incorporating PEG into the formulation, therefore, potentially overcoming the side effects attributed to PEG.

Compared with PDA-only NPs, hybrid systems of PDA/liposomes offer a series of advantages. It is possible to easily obtain PDA nanoparticles with uniform size distributions without resorting to complicated procedures and avoiding the use of organic solvents and other poorly biocompatible reagents. Compared with simple PDA nanoparticles, PDA-coated liposomes take advantage of the compartmentalization offered by the lipid vesicles with the possibility of expanding the transport and delivery capabilities of the PDA particles. Liposomes can also be used as a sacrificial templating agent to obtain hollow PDA particles with interesting application properties. Furthermore, recent studies have shown that the hemolytic activity and cytotoxicity of PDA depend on the thickness of the polymer. Therefore, the use of liposomes covered with thin layers of PDA would allow, compared with full PDA particles, the acquisition of safer systems for biomedical applications.

## 6 Future Outlook

Beyond the presented works, a wide variety of applications of PDA-modified liposomes is still imaginable. For instance, the ROS scavenger capabilities of Lipo@PDA have yet to be explored, as well as the possibility of treating the PDA shell to make it fluorescent and exploit these systems for bioimaging purposes. Notably, the presence of a PDA coating around a liposome introduces a new drug storage site. In fact, liposomes as such can encapsulate bioactive compounds into the aqueous core and/or the lipid bilayer. In Lipo@PDA, the polymeric shell could be actively employed to transport further compounds of interest. It could be useful to deliver more cargoes simultaneously or to span their release over time, thereby promoting first the release of the compounds adsorbed onto the outer polymeric shell and then the one of the compounds entrapped in the internal structure of the vesicle.

However, to translate the use of Lipo@PDA to the clinic practice, multiple aspects should be still considered. There is a need for proper characterization methods for the evaluation of the safety, pharmacokinetics, and efficacy of these nanosystems. For example, the effects induced in the organism after the administration of Lipo@PDA vesicles *in vivo* have never been objects of study and should be further investigated with particular attention to immunogenic responses triggered after multiple dose administration. In addition, once these systems are administered into the body, targeting agents can be shielded because of the formation of the protein corona, which results in reduced therapeutic efficacy.

Moreover, as for the other types of nanosystems, major problems are related to the need to develop reproducible and cost-effective production and scale-up methods. It is known that by moving to large-scale production from laboratory-scale quantities, variations in the physicochemical properties of the particles may occur, thereby resulting in altered biological behavior.
